# Assessment of partograph knowledge and associated factors among obstetric care providers in West Cameroon: a cross-sectional study

**DOI:** 10.11604/pamj.2026.53.92.50810

**Published:** 2026-02-20

**Authors:** Armand Duclaire Kemo Djimeli, Bruno Kenfack, Jerome Ateudjieu

**Affiliations:** 1 Department of Public Health, Faculty of Medicine and Pharmaceutical Sciences, University of Dschang, Dschang, Cameroon,; 2 Department of Obstetrics and Gynecology and Mother Health, Faculty of Medicine and Pharmaceutical Sciences, University of Dschang, Dschang, Cameroon

**Keywords:** Partograph, knowledge, obstetric care providers, West, Cameroon

## Abstract

**Introduction:**

the partograph is a graphical record of the progression of labour and the main maternal and fetal parameters over time. Partograph allows early detection of abnormalities in the progression of labour, such as obstructed labour, which was responsible for 8.3% of maternal deaths and 39.7% of stillbirths in sub-Saharan African countries. The objective of this study was to assess knowledge and identify factors associated with a good level of knowledge among obstetric care providers regarding the partograph.

**Methods:**

this was a cross-sectional study conducted between February 1^st^ and June 30^th^, 2024. The study was conducted in 14 Health Districts, including urban-rural and rural Health Districts selected among the 20 Health Districts in the West Region of Cameroon, using a stratified random sampling technique. Data were collected using a semi-structured self-administered and pre-tested questionnaire administered to obstetric care providers encountered during a monthly coordination meeting of healthcare facilities in the study´s Health Districts. Descriptive statistics, univariable and multivariable logistic regression analysis were performed. We determined the adjusted odds ratios with a 95% confidence interval, and a P-value less than 0.05 was considered statistically significant.

**Results:**

a total of 373 obstetric care providers participated in the study, with an average age of 39.6 ± 10.6 years; 64.6% (241) were women, 64.3% (240) were nurses, and 79.1% (295) worked in Health Centers. The proportion of obstetric care providers with good knowledge of the partograph was 59.8%. Working in a District Hospital/Medical Center aOR: 2.76, 95% CI 1.24-6.87; p = 0.019, being a midwife/general practitioner aOR: 5.41, 95% CI 1.51- 26.8; p = 0.018, working in a health facility located in a rural Health District aOR: 1.71, 95% CI 1.05-2.81; p = 0.032, and having 6-10 years of service aOR: 6.03, 95% CI 1.22-35.4; p = 0.031 were factors associated with good level of knowledge about the partograph. In addition, 94.1% (351) obstetric care providers expressed the need for additional training on the partograph.

**Conclusion:**

less than two-thirds of participants had a good knowledge of the partograph. We recommend conducting interventions aimed at capacity building and the systematic use of partograph by obstetric care providers in general, and those working in Health Centers in particular; ensure that work teams are formed by pairing less experienced obstetric care providers with those who have more years of service and increase the recruitment of midwives in different health facilities.

## Introduction

In 2023, approximately 260,000 maternal deaths occurred worldwide, with the highest maternal mortality ratio recorded in sub-Saharan Africa, at 454 maternal deaths per 100,000 live births, compared to 11 maternal deaths per 100,000 live births in Europe and North America [1]. In Cameroon, the maternal mortality ratio fell from 678 deaths per 100,000 live births in 2000 to 258 maternal deaths per 100,000 live births in 2023, a reduction of approximately 62% [1]; however, efforts are still needed to achieve the sustainable development goal set by the World Health Organization, which aims for a maternal mortality rate of less than 70 maternal deaths per 100,000 live births by 2030 [2].

A study conducted by the World Health Organization in South Asia found that the partograph is necessary for labour management and that its proper use reduces the proportion of labour requiring increased contractions (from 20.7% to 9.1%), prolonged labour (from 6.4% to 3.4%), emergency cesarian section rates (from 9.9% to 8.3%), and stillbirths (from 0.5% to 0.3%) [3,4]. In Ethiopia, the partograph allows early detection of abnormalities in labour progression, such as obstructed labour, which was responsible for 8.3% of maternal deaths and 39.7% of stillbirths [5]. His utilization remains sub-optimal, a systematic review and meta-analysis revealed that the pooled prevalence of systematic use of the partograph in sub-Saharan Africa was 51.17% [6]. Studies conducted on the partograph have found that the proportion of obstetric care providers with a good level of knowledge about the partograph was: 49.1% in Ethiopia [7]; 59% in Nigeria [8]; 60% in Kenya [9].

A limited number of studies have been conducted on the partograph in Cameroon, showing that 21.3% to 62% of obstetric care providers in the North-West and South-West regions [10-12] and 35% in the Central region [13] had a good level of knowledge about the partograph. Thus, this study aimed to determine the level of knowledge and factors associated with good partograph knowledge among obstetric care providers in West Cameroon in order to provide appropriate strategies for optimal labour management and monitoring and to ensure safe maternity practices.

## Methods

**Study design, period and setting:** this was a cross-sectional study conducted from February 1^st^ to June 30^th^, 2024 in the West, which is one of Cameroon's ten regions. The West Region has Bafoussam as its capital and comprises 20 Health Districts, including eight urban-rural Health Districts: Foumban, Mifi, Dschang, Mbouda, Malantouen, Bafang, Bangangte, and Foumbot, as well as 12 rural Health Districts: Penka-Michel, Batcham, Baham, Bandja, Santchou, Bamendjou, Bangourain, Bandjoun, Galim, Massangam, Kekem, and Kouoptamo.

**Study population:** the study population consisted of all obstetric care providers working part-time or full-time in maternity wards in public or private health facilities in 14 Health Districts selected using a stratified random sampling technique among the 20 Health Districts in the West, including seven urban-rural Health Districts: Foumban, Mifi, Dschang, Mbouda, Malantouen, Bafang, Bangangte, and seven rural Health Districts: Penka-Michel, Batcham, Baham, Bandja, Santchou, Bamendjou, and Bangourain.

**Inclusion and exclusion criteria:** all general practitioners, midwives, nurses, and other healthcare providers offering care to women in labour who attended a monthly district coordination meeting of the study health districts were included in the study. Obstetric care providers who did not give their informed consent were excluded from the study.

**Sample size estimation:** to calculate the sample size, we used a single population proportion formula, n = (Zα/2)^2^ P(1-P)/d^2^ [14]; considering that the proportion of obstetric care providers with a good level of knowledge about the partograph is P=44.8% according to the study conducted by Wakeshi and Molla [15]; with a 95% confidence interval, Z=1.96 and a margin of error d= 5%. N=(1.96)^2^ x (0.448)x(1-0.448)/(0.05)^2^=0.247296x3.8416/0.0025=380. Furthermore, considering a non-response rate of 5%, we obtain a sample size of 399.

**Data collection:** data were collected using a semi-structured, self-administered questionnaire developed on the basis of previous studies [10,14]. This questionnaire was pre-tested by administering it to obstetric care providers in a Health District not included in the study, then adopted without modification. The data collected covered several variables, including the dependent variable: partograph knowledge and independent variables: socio-demographic characteristics, factors related to obstetric care providers and factors related to health facilities.

### Operational Definitions

***Partograph knowledge:*** level of knowledge of obstetric care providers on the partogram assessed using a questionnaire scored out of 30 points in total. A correct answer corresponds to 1 point and an incorrect answer corresponds to 0 points.

***Good partograph knowledge:*** proportion of obstetric care providers with a partograph knowledge score between 21 and 30.

***Fair partograph knowledge:*** proportion of obstetric care providers with a partograph knowledge score between 11 and 20.

***Poor partograph knowledge:*** proportion of obstetric care providers with a partograph knowledge score between 0 and 0.

***Partograph utilisation:*** systematic use of the partograph by obstetric care providers to monitor women in labour. To determine the proportion of obstetric care providers who systematically use the partograph, two questions were asked: Have you used the partograph in the six months before the study? Yes or No; for those who answered yes, how often do you use the partograph: always, sometimes, or rarely. We thus determined the proportion of obstetrics care providers who use the partograph systematically (always) and the proportion of those who do not use the partograph systematically (sometimes, rarely, or never).

***Obstetric care providers:*** healthcare providers such as general practitioners, nurses, midwives, and other healthcare providers who monitor labour progress and offer delivery services.

**Statistical analysis:** the collected data were entered into CsPro software version 7.3, then exported to R software version 4.3.3 for analysis. Descriptive statistical analysis was performed, consisting of calculating the mean with standard deviation and frequency; univariable and multivariable logistic regression analysis were also performed to determine the association between the dependent variable and the independent variables; the results were presented in tables and figure. Variables with a p-value less than 0.2 in the univariable logistic regression analysis were included in the multivariable logistic regression analysis model. The odds ratio with a 95% confidence interval was calculated to determine the existence and strength of the association, and a p-value less than 0.05 was considered statistically significant.

**Ethical considerations:** authorization from the Regional Delegate for Public Health in the West was obtained before the implementation of this study. In addition, ethical clearance was issued by the Regional Ethics Committee for Human Health Research in West Cameroon, number 1018/27/12/2023/CE/CRERSH-OU/VP, dated December 27, 2023, before the study began. The informed consent of the participants was obtained before distributing the questionnaires. Confidentiality was ensured by the anonymity of the questionnaire forms. The study adhered to the principles of the Declaration of Helsinki.

## Results

**Socio-demographic characteristics of obstetric care providers:** a total of 373 obstetric care providers were enrolled in the study, making a response rate of 93.5%. Table 1 presents the socio-demographic characteristics of the study participants; the average age of participants was 39.6 ± 10.6 years. More than half of the obstetric care providers (64.6%) were women, and 64.3% were nurses. Two hundred and fifty-eight (69.2%) obstetric care providers were married; two hundred and ninety-five (79.1%) worked in health centers and almost three-quarters (71.4%) had been practising for six years or more.

**Table 1:** socio-demographic characteristics of obstetric care providers, 2024 (N=373)

Variables	Frequency	Percent
**Sex**		
Male	132	35.4%
Female	241	64.6%
**Age (in years)**		
20-29	86	23.1%
30-39	97	26.0%
40-49	108	29.0%
>=50	82	22.0%
**Marital status**		
Married	258	69.2%
Single*	115	30.8%
**Professional qualification**		
Nurse assistant	109	29.2%
Nurse	240	64.3%
Midwife	19	5.1%
General practitioner	5	1.3%
**Religion**		
Christian	301	80.7%
Muslim	52	13.9%
Others	20	5.4%
**Place of work**		
Health center	295	79.1%
District Hospital / Medical Center	47	12.6%
Clinic	31	8.3%
**Location of health facility**		
Urbano-Rural	262	70.2%
Rural	111	29.8%
**Year of service**		
<1	8	2.1%
1-5	99	26.5%
6-10	63	17.0%
>10	203	54.4%

* Single = Unmarried, Divorced, Widow/Widower

**Table 2:** detailed knowledge of obstetric care providers about the partograph, 2024 (N=373)

Variables	Correct		Incorrect	
	**n**	**%**	**n**	**%**
The partograph is a complex tool with a pictorial overview of labour for use by midwives	233	62.5%	140	37.5%
The partograph is a chart for monitoring labour by doctors	274	73.5%	99	26.5%
The partograph is a simple graphic recording of labour and salient conditions of the mother and fetus against time in hours	357	95.7%	16	4.3%
The partograph will reduce maternal morbidity and mortality	303	81.2%	70	18.8%
The partograph will reduce neonatal morbidity and mortality	268	71.8%	105	28.2%
The partograph is only performed in women with pre-eclampsia/eclampsia and primiparous	346	92.8%	27	7.2%
The partograph allows early detection of abnormalities in labour progression	336	90.1%	37	9.9%
The partograph should be started when cervical dilation is 4 centimetres	344	92.2%	29	7.8%
The partograph should be started when the woman feels pains like uterine contractions	343	92%	30	8%
The start of filling the partograph is conditioned by the rupture of the water bag	357	95.7%	16	4.3%
The first recording of cervical dilation on the partogram should be made on the vertical line	159	42.6%	214	57.4%
The first recording of cervical dilation on the partogram should be made on the alert line	94	25.2%	279	74.8%
The graph on the partograph should fall to the left of the alert line	154	41.3%	219	58.7%
The graph or plot of labour progress should ideally fall between the alert line and the action line	144	38.6%	229	61.4%
The graph or plot of the labour progress should ideally fall to the right of the action line	199	53.4%	174	46.6%
When the cervical dilation curve moves to the right of the alert line, this may indicate slow labour progression.	198	53.1%	175	46.9%
You require 10 minutes to assess the adequacy of contractions effectively	260	69.7%	113	30.3%
Minimum duration of a strong contraction is 40 seconds	245	65.7%	128	34.3%
The progress of labour is assessed by the degree of cervical dilatation and descent of the presenting part	312	83.6%	61	16.4%
Labour is prolonged when it lasts more than 12 hours	226	60.6%	147	39.4%
Knowledge on assessment inferred from the partograph				
Prolonged labour	298	79.9%	75	20.1%
Obstructed labour	307	82.3%	66	17.7%
Inefficient uterine contraction	286	76.7%	87	23.3%
Satisfactory progress of labour	294	78.8%	79	21.2%
Poor progress of labour	289	77.5%	84	22.5%
Need to refer the woman to a hospital	310	83.1%	63	16.9%
Suspected foetal distress	300	80.4%	73	19.6%
Need for labour augmentation	189	50.7%	184	49.3%
Need for Caesarean delivery	274	73.5%	99	26.5%
Dehydration in the mother	67	18%	306	82%

**Detailed knowledge of the partograph:** Table 2 shows obstetric care providers' knowledge of the partograph in West Cameroon. Almost all (95.7%) participants identified the correct definition of the partograph, noting that it is a tool for graphically recording the progress of labour and the main maternal and fetal parameters over time in hours. Knowledge of the partograph as a prevention tool showed that 81.2% and 71.8% of obstetric care providers stated that the use of the partograph reduces maternal morbidity and mortality, and neonatal morbidity and mortality, respectively. Only 25.2% of participants responded that the first recording of cervical dilation should be made on the alert line. Obstetric care providers' knowledge of the graphical representation of normal labour progression was low, with only 41.3% correctly stating that, in normal labour progression, the graph of labour progress should fall to the left of the alert line. Less than three-quarters (69.7%) agreed that it takes 10 minutes to assess the adequacy of uterine contractions, and only 60.6% correctly stated that prolonged labour is labour that lasts more than 12 hours. Regarding the different diagnoses that can be made using a partograph, only 50.7% recognized that it can be used to assess the need to increase uterine contractions.

**Figure 1:**
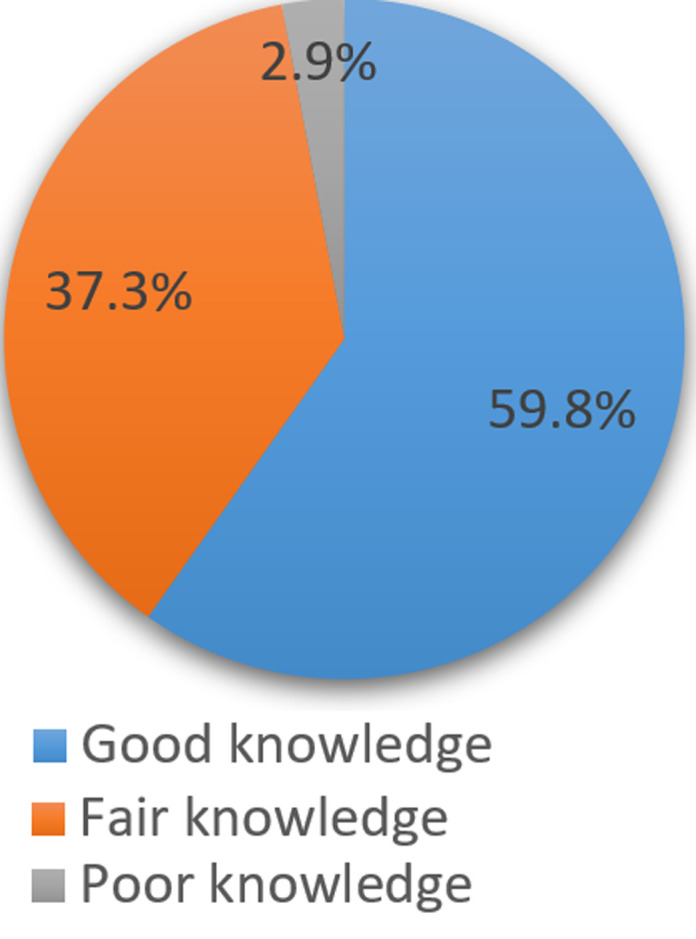
overall knowledge of obstetric care providers about the partograph, 2024 (N=373)

**Overall knowledge of the partograph:** overall, obstetric care providers' knowledge scores on the partograph ranged from 2 to 30, with an average score of 20.7 ± 4.6. Figure 1 shows that 59.8% of obstetric care providers had a good level of knowledge about the partograph.

**Utilization of partograph:** the magnitude of partograph use was 72.1%, 95% CI: 67.3 - 76.6; that is, 269 (72.1%) of obstetric care providers systematically used the partograph, while 71 (19%) used it sometimes, 14 (3.8%) rarely used the partograph, and 19 (5.1%) reported never using the partograph.

**Factors associated with good knowledge on the partograph:** Table 3 explores factors associated with a good level of knowledge of the partograph. Thus, the probability of having good knowledge of the partograph was 5.41 times higher among midwives and general practitioners than among nursing assistants aOR: 5.41, 95% CI 1.51- 26.8; p = 0.018. In addition, the probability of having good knowledge of the partograph was 2.76 times higher among obstetric care providers working at the District Hospital/Medical Center compared to those working at the Health Center aOR: 2.76, 95% CI 1.24-6.87; p = 0.019. The probability of having good knowledge of the partograph was 1.71 times higher among obstetric care providers working in a healthcare facility located in a rural Health District compared to those working in a healthcare facility located in an urban-rural Health District aOR: 1.71, 95% CI 1.05-2.81; p = 0.032. In addition, the probability of having good knowledge of the partograph was six times higher among obstetric care providers with 6-10 years of professional experience compared to those who had been practising for less than a year aOR: 6.03, 95% CI 1.22-35.4; p = 0.031. In addition, 94.1% (351) of obstetric care providers expressed the need for additional training on the partograph.

**Table 3:** factors associated with good partograph knowledge among obstetric care providers, 2024 (N=373)

Variables	Good partograph knowledge			
	Unadjusted OR (95% CI)	P-value	Adjusted OR (95% CI)	P-value
**Sex**				
Male	1.57 (1.01-2.46)	0.046	1.49 (0.93-2.42)	0.10
**Professional qualification**				
Nurse	1.55 (0.98-2.45)	0.059	1.56 (0.95-2.57)	0.079
Midwife/General practitioner	6.87 (2.21-30.30)	0.003	5.41 (1.51- 26.8)*	0.018
**Type of institution**				
District Hospital /Medical Centre	3.63 (1.72-8.63)	0.001	2.76 (1.24-6.87)*	0.019
Clinic	0.70 (0.33-1.47)	0.3	0.66 (0.30-1.44)	0.3
**Location of health facility**				
Rural	1.43 (0.91-2.29)	0.13	1.71 (1.05-2.81)*	0.032
**Year of service**				
1-5	1.92 (0.45-9.77)	0.4	3.03 (0.64-17.00)	0.2
6-10	4.17 (0.93-22.10)	0.068	6.03 (1.22-35.40)*	0.031
>10	2.51 (0.60-12.50)	0.2	3.91 (0.85-21.60)	0.088
**Routine utilization of partograph**				
Yes	1.74 (1.10-2.76)	0.017	1.55 (0.95-2.53)	0.08

OR = Odd Ratio, CI= Confidence Interval , * = statistically significant at 95% confidence interval

## Discussion

The objective of this study was to determine the level of knowledge and factors associated with a good level of knowledge of the partograph among obstetric care providers in West Cameroon; the results show that 59.8% of obstetric care providers had good knowledge about the partograph in West Region, Cameroon. In addition, professional qualification aOR: 5.41, 95% CI 1.51- 26.8; p = 0.018, type of institution aOR: 2.76, 95% CI 1.24-6.87; p = 0.019, location of health facility aOR: 1.71, 95% CI 1.05-2.81; p = 0.032 and number of years´ service aOR: 6.03, 95% CI 1.22-35.4; p = 0.031 were significantly associated with good knowledge of the partograph. In addition, the level of systematic partograph utilisation was 72.1%.

Our study revealed that less than two-thirds of obstetric care providers (59.8%) had overall good knowledge of the partograph; our result falls in line with the study conducted by Agan *et al*. who found that 58.3% of obstetric care providers had a good level of knowledge about the partograph [16]; 53.7% according to Mezmur *et al*. in Ethiopia [14]; 61.8% in the study conducted by Verla *et al*. in Cameroon [12]; however, the proportion of obstetric care providers with a good level of knowledge about the partograph was higher (78%) in the study conducted in Ghana [17], this can be explained by the fact that their study population consisted only of midwives, who have received basic training in obstetric care. In addition, detailed knowledge of partograph among obstetrics care providers revealed that only 25.2% of our study participants knew that the first measurement of cervical dilation should be taken at the alert line. Furthermore, less than half of obstetric care providers, 41.3% stated that the graph of labour progress should ideally fall to the left of the alert line; this result is similar to that obtained by Sama *et al*. 42.3% in Northwest and Southwest Cameroon [10], revealing that some obstetric care providers may be filling out the partograph with limited ability to interpret it; Agan *et al*. found that only 30% of obstetric care providers were able to correctly explain the function of the action line [16]; these authors reported that it could contribute to an increase in the incidence of prolonged labour, as the action line generally serves as the basis for intervention during labour. Our study also revealed that less than two-thirds of respondents (60.6%) answered that labour is prolonged when it lasts more than 12 hours; this proportion was 62% in the study conducted in Northwest and Southwest Cameroon [10]. Regarding knowledge of the characteristics of normal labour, approximately one-third (30.3%) did not know that 10 minutes are needed to assess the adequacy of uterine contractions, and 34.3% did not know that the minimum duration of a strong contraction is 40 seconds; in the study conducted by Agan *et al*. 50% of respondents did not know that 10 minutes are needed to assess the adequacy of contractions, and 59.8% of obstetric care providers did not recognize that the minimum duration of a strong contraction is 40 seconds [16]. Although 76.7% of respondents acknowledged knowing how to identify ineffective uterine contractions using a partograph, only 50.7% of respondents reported being able to use a partograph to assess the need to increase labour. These results highlight the need to implement strategies that will raise the level of knowledge of obstetric care providers regarding the partograph. This is how 94.1% (351) obstetric care providers expressed the need for additional training on the partograph.

The proportion of obstetric care providers who systematically use the partograph was 72.1%, which is higher than the pooled prevalence of systematic use of the partograph in sub-Saharan Africa, 51.17% [6]; however, further efforts are needed, as the World Health Organization recommends the use of the partograph for monitoring all women in labour for a safe motherhood [3]. This therefore, highlights the need to implement interventions that will encourage obstetric care providers to systematically use the partograph to monitor women in labour.

Our study revealed that midwives and general practitioners had 5.41 times more knowledge of the partograph than nursing assistants; this result is similar to those of studies conducted in Nigeria and South Africa [18,19]. Our result could be explained by the fact that midwives have received basic training that is more focused on reproductive health compared to nurses and nursing assistants during pre-service; thus, they are better equipped to use the partograph to monitor women in labour. This result calls on health authorities to advocate for and/or recruit more midwives in different health facilities to improve the effectiveness of labour monitoring using the partograph. Also, obstetric care providers who have been working at District Hospital/Medical Center were 2.76 times more likely to have good knowledge on partograph compared to those who have been working at health centers; this result could be explained by the presence of doctors in District Hospitals and Medical Centers, unlike Health Centers, which do not have them. This implies the need for more intensive capacity building for obstetric care providers working in health centers on the partograph. This result relates to a study conducted by Mezmur *et al*. which shows that the place of work was significantly associated with a good level of knowledge on partograph [14]. In addition, obstetric care providers who have been working in health facility located rural Health District were 1.71 times more likely to have good knowledge on partograph compared to those who have been working at urban-rural Health District; this result could be explained by the fact that there are probably more Health Centers in urban-rural Health Districts than in rural Health Districts; in addition, we observed above that obstetric care providers working in District Hospitals and Medical Centers had 2.76 times more knowledge about the partograph than those working in Health Centers. Obstetric care providers who had 6-10 years of service were 6 times more likely to have good knowledge on partograph than obstetric care providers with less than one year of service. This result implies that when forming work teams in health facilities, a less experienced obstetric care provider should be paired with a more experienced obstetric care providers to optimally monitor labour using the partograph, which is a decision-making tool, enabling early detection of abnormalities in labour progression, such as obstructed labour, which was responsible for 8.3% of maternal deaths and 39.7% of stillbirths in a study conducted in sub-Saharan African country [5].

Since information was obtained from respondents through self-administered questionnaires, rather than observation, there is a tendency to social desirability bias, whereby respondents may be inclined to give answers that they consider desirable in the eyes of researchers. To reduce the response bias, the aim of the study was discussed with respondents in order to obtain a genuine response. This study was conducted in 14 Health Districts, including both urban-rural and rural Health Districts, randomly selected among the 20 Health Districts in the West, which is a strength of the study. In addition, the sample size was large, which made it possible to explore the determinants of a good level of knowledge about the partograph; furthermore, the study participants were obstetric care providers from both public and private healthcare facilities; thus, the findings can be generalised to the West region of Cameroon. It would be relevant to explore obstetric care providers' knowledge of the partograph in other regions of the country.

## Conclusion

This study revealed that less than two-thirds of obstetrics care providers had an overall good knowledge about the partograph in the West region of Cameroon. Professional qualification, type of institution, location of health facility and year of service were the variables found to be significantly associated with good knowledge of the partograph. The level of partograph utilisation was 72.1%, which is lower than WHO recommendations. We recommend conducting interventions aimed at capacity building and the systematic use of partograph by obstetric care providers in general, and those working in health centres in particular; ensure that work teams are formed by pairing less experienced obstetric care providers with those who have more years of service and increase the recruitment of midwives in different health facilities.

### What is known about this topic

- The proper use of the partograph reduces the proportion of labour requiring increased contractions (from 20.7% to 9.1%), prolonged labour (from 6.4% to 3.4%), emergency cesarian section rates (from 9.9% to 8.3%), and stillbirths (from 0.5% to 0.3%);
- The pooled prevalence of systematic use of the partograph in sub-Saharan Africa is 51.17%, which is almost half the World Health Organization's recommendation;
- The proportion of obstetric care providers with a good level of knowledge about the partograph varied between 21.3% and 62% in the North-West, South-West and Central regions of Cameroon.

### What this study adds

- Less than two-thirds (59.8%) of obstetric care providers were found to have good knowledge of the partograph, which can affect the quality of partograph utilization;
- The level of partograph utilisation in the study was 72.1%, 95% CI 67.3 - 76.6, which is low compared to the World Health Organization recommendation;
- The factors associated with a good level of knowledge on partograph were: working in the District Hospital/Medical Center, being a midwife/general practitioner, working in a health facility located in a rural Health District and having 6-10 years of service.
